# Disbiome database: linking the microbiome to disease

**DOI:** 10.1186/s12866-018-1197-5

**Published:** 2018-06-04

**Authors:** Yorick Janssens, Joachim Nielandt, Antoon Bronselaer, Nathan Debunne, Frederick Verbeke, Evelien Wynendaele, Filip Van Immerseel, Yves-Paul Vandewynckel, Guy De Tré, Bart De Spiegeleer

**Affiliations:** 1 Drug Quality and Registration (DruQuaR) Group, Faculty of Pharmaceutical Sciences, Ghent University, Ottergemsesteenweg 460, B-9000 Ghent, Belgium; 2 Department of Telecommunications and Information Processing, Faculty of Engineering and Architecture, Ghent University, Sint-Pietersnieuwstraat 41, B-9000 Ghent, Belgium; 3 Department of Pathology, Bacteriology and Poultry Diseases, Faculty of Veterinary Sciences, Ghent University, Salisburylaan 133, B-9820 Merelbeke, Belgium; 4 Department of Internal Medicine, Hepatology Research Unit; Faculty of Medicine and Health Sciences, Ghent University, De Pintelaan 185, B-9000 Ghent, Belgium

**Keywords:** Dysbiosis, Database, MedDRA, Health status

## Abstract

**Background:**

Recent research has provided fascinating indications and evidence that the host health is linked to its microbial inhabitants. Due to the development of high-throughput sequencing technologies, more and more data covering microbial composition changes in different disease types are emerging. However, this information is dispersed over a wide variety of medical and biomedical disciplines.

**Description:**

Disbiome is a database which collects and presents published microbiota-disease information in a standardized way. The diseases are classified using the MedDRA classification system and the micro-organisms are linked to their NCBI and SILVA taxonomy. Finally, each study included in the Disbiome database is assessed for its reporting quality using a standardized questionnaire.

**Conclusions:**

Disbiome is the first database giving a clear, concise and up-to-date overview of microbial composition differences in diseases, together with the relevant information of the studies published. The strength of this database lies within the combination of the presence of references to other databases, which enables both specific and diverse search strategies within the Disbiome database, and the human annotation which ensures a simple and structured presentation of the available data.

## Background

For many years, it has been believed that the human body has a microbial cell content which exceeds the total amount of human somatic cells by tenfold [1]. More recently, it has been estimated that this ratio between microbial and human cells is closer to 1:1 [2]. The collection of these microorganisms is termed ‘microbiota’ and the collective genomes of all the microorganisms of these microbiota are defined as the microbiome [3]. The main part of this microbiota is situated in the gut, in which the numbers and complexity increases from the stomach to the colon [4, 5]. Other anatomical sites which have their own microbiome are the lungs, skin, vagina, eyes, placenta, ear, oral cavity and sino-nasal compartment. The composition of the microbiome varies by anatomical site (e.g. between the gut and skin), between individuals and even over time [6, 7]. The microbiome composition can change due to factors such as dietary changes including pre- and probiotic use, antibiotic and other medicine use, age or disease and is moreover dynamic on its own [8, 9] . The microbiota composition and its correlation with health/disease is thus considered a multifactorial process. An active lifestyle can influence the gut microbiota composition, enhancing diversity and promoting bacterial communities associated with healthy individuals, which tend to be dominated by species such as *Faecalibacterium prausnitzii, Roseburia hominis* and *Akkermansia muciniphila* [10, 11]. Moreover, the mode of delivery has a major influence on the microbiome composition of the new born. After vaginal delivery, the baby’s microbiome resembles the mother’s genital and gastrointestinal tract while bacteria of the skin appear to be more abundant after caesarian section [12]. Several intrinsic and extrinsic factors influence the development and variation of bacteria in infants. Genetics and epigenetics, environmental factors like geography and diet (breastmilk or formula fed) all affect development of the microbial population [13, 14]. However, a lot of questions concerning the development of the fetus and neonate microbiome are still open [15].

While it was initially thought that microbes are mainly commensals whose only benefit is controlling the population of pathogenic bacteria, there is compelling evidence that the gut microbiome also has health-influencing effects, playing roles in *i.a.* digestion, inflammation, intestinal integrity and development of the immune system [16]. Production of microbial metabolites are a key driver in these processes. Host health thus appears to be closely related to a homeostatic balanced relationship with the microbial inhabitants. Several diseases are associated with an altered microbiota composition, such as obesity [17], diabetes [18], Crohn’s disease [19], ulcerative colitis [20], autism [21], bacterial vaginosis [22] and psoriasis [23]. These alterations are not limited to the location of the disease. As an example, alterations of the gut microbiota are seen in a variety of central nervous disorders, supporting the presence of a gut-brain axis [24, 25]. At this point, it remains to be elucidated whether the observed microbiota differences in various disease states are a symptom of the disease or have a more causal effect [6]. Suppressing clinical dysbiosis and restoring the altered microbiome to a ‘healthy’ microbiome can be a potential approach to improve host health. Potential therapeutic options include narrow spectrum antibiotics, probiotics, prebiotics, dietary interventions and fecal transplantation [16].

Different microbiology databases for research are available. There are databases covering different microbial subjects such as genomic resources (e.g. IMG) [26], protein families (e.g. Pfam) [27], diversity (e.g. SILVA) [28], model organisms (e.g. EcoCyc) [29], pathogenesis (e.g. EuPathDB) [30], transport and metabolism (e.g. TCDB) [31] and signal transduction and gene regulation (e.g. MiST) [32]. However, a database covering microbiome differences in different disease states is, to our knowledge, currently missing. Seen the exploding data of microbiome alterations in different disease states, we present the Disbiome database, collecting and organizing this information (https://disbiome.ugent.be). Disbiome encompasses microbiome differences between patients and controls together with the used detection method and sample type. This database differs from other comparative tools such as MG-RAST as it presents comparisons between patient and control data in a clear and concise manner to the broader audience in a programmatically accessible way using the JSON export format [33]. Disbiome can be valuable for every researcher in the field of microbiology to rapidly and easily find bacterial species possibly correlated to specific diseases to further explore its mode of actions and interaction mechanisms with the host. It can speed up translational research in microbiome modulations (by either probiotics, prebiotics and microbiota transplantation) for treating a variety of diseases. In addition, it can serve as a new disease classification system based on microbiome changes. Currently, the database includes over 190 different diseases and 800 different organisms. Changes in organisms are detected by over 25 different detection methods (e.g. qPCR, next-generation sequencing,…) in 50 different sample types (e.g. faeces, skin swabs, tissue biopsies,…).

## Construction and content

**Fig. 1:**
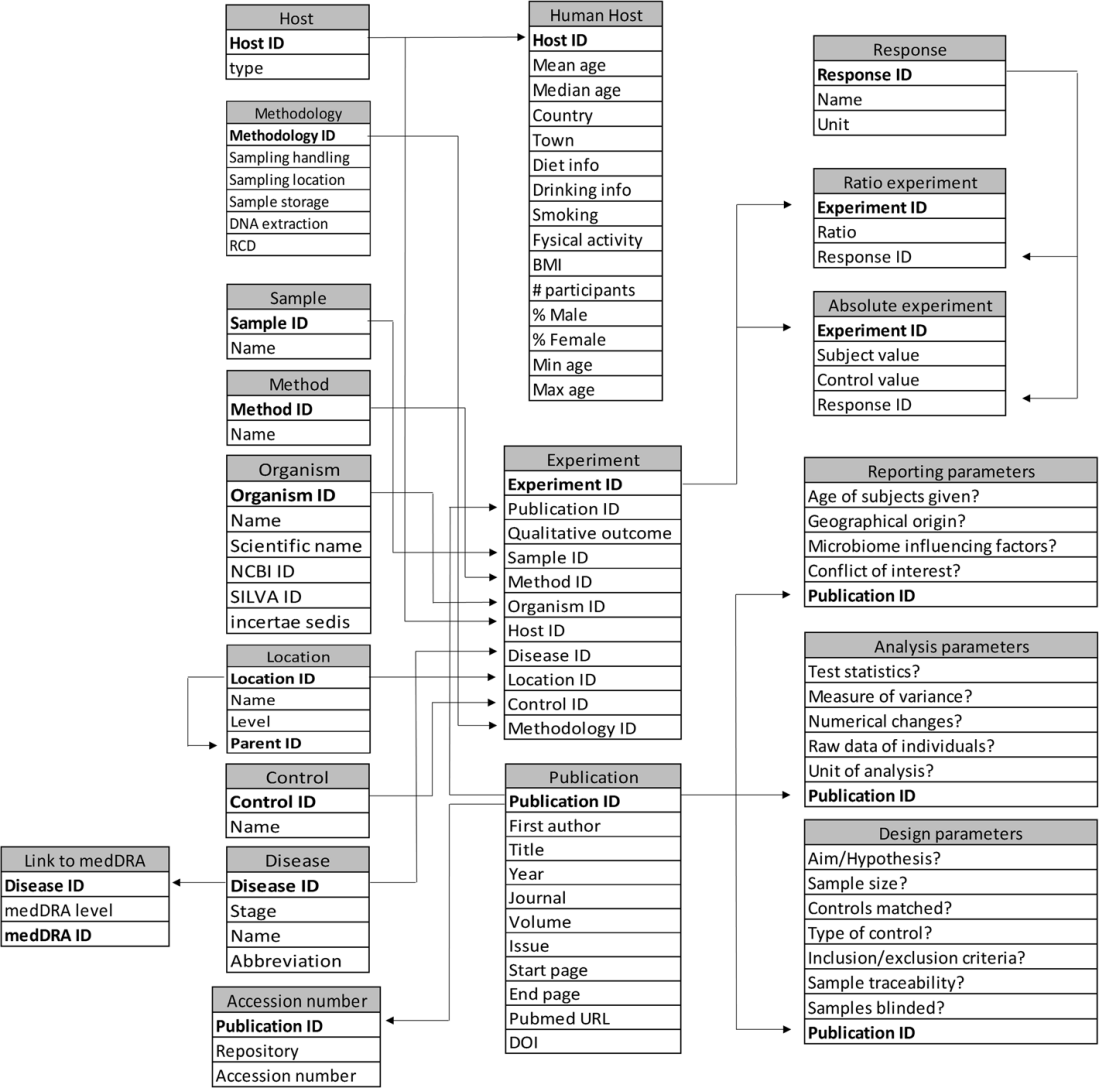
Database scheme. RCD = Reference Classification Database

To list all relevant data, a relational database was constructed [34]. A relational database separates the design of the data from its physical representation. Data are designed as tables where the rows represent distinct entities and the columns represent various attributes of those entities [35]. The schematic database model is given in Fig. 1. This visual representation represents the structure of the database. A block represents an entity type, where the table’s columns represent the entity type’s different attributes. Such entity type can have an unlimited number of entities. In the physical database, each entity type translates to a table where each attribute represents a column of that table and each entity is represented by a row. The central entity in the Disbiome database is ‘Experiment’, representing the microbiome difference between a patient and control. Every experiment has a qualitative outcome (elevated or reduced) and is related to different parameters. The experiment is linked to the appropriate publication (Publication ID), showing a microbial (Organism ID) difference between a sample (Sample ID) of a patient (Disease ID, Host ID) and a control subject (Control ID) using a specific detection method (Method ID). This sample originates from a specific location (Location ID). The microbial difference can be presented by absolute quantities between the patient and control or by a ratio. In the case of absolute data, a specific response unit, dependent on the used detection method, is present (Response ID). When absolute data is not available, the microbial differences are only presented by the qualitative outcome. Every host and publication is linked to different host and publication parameters respectively and diseases are linked to their classification in the Medical dictionary for Regulatory Activities (MedDRA). The storage of Disbiome was implemented using PostgreSQL, an open source database. This is accessed by LimeDS, a framework developed at Ghent University, providing a web service with which the website (disbiome.ugent.be) communicates [36]. Several search options are present in the Disbiome database. Organisms, diseases and detection methods can be used as queries and will give an overview of the experiments related to this organism, disease or detection method. From this overview page, detailed information about the experiment can be obtained.

Literature data was collected by using the search engine PubMed, covering the period 2009–2018. The search queries [(‘microbiota’ OR ‘microbiome) AND (‘health’ OR ‘disease’)] and [microbiome alterations] were used. This search gave approximately 20.000 publications. Based on title (exclusion of duplicates, reviews, animal studies, studies on effect of medication, symposium/meeting abstracts as well as non-English language papers), only around 1.000 publications were withheld and based on the abstract (only case-control studies) around 500 publications were found to be suitable for insertion in the database. The obtained literature was processed manually and all relevant information was put in the database. Currently only human data is incorporated but information of other species (e.g. mice, rats,…) will be included as well. The database will be updated manually every 3 months. An automated updating system is being developed for further versions of the database. Additionally, authors of publications can inform us of missing manuscripts by using the ‘Submission’ link.

## Utility and discussion

Our main objective was to construct a database giving a clear and rapid overview of all bacterial species which are differentially present in a particular disease.

### Experiment

The experiment section is the central table of the database. It contains all the relevant information about the experiment (disease, detected organism, quantitative data of the patient and control, control type, response type and detection method), all other data is linked to this experiment as well (e.g. publication info, host details, methodological details and sample type). The sample type is of great importance because it can influence the detected microbiome composition. Tedjo et al. demonstrated a higher microbial diversity using fecal swabs compared to stool samples in the same subjects [37].

### Disease

The diseases are classified using the classification of the Medical Dictionary for Regulatory Activities (MedDRA). It is developed by the International Council for Harmonization of Technical Requirements for Pharmaceuticals for Human Use (ICH) to provide a single standardized international medical terminology to facilitate sharing of regulatory information for medical products used by humans. MedDRA consists of a five-level structural hierarchy, arranged from very specific to very general levels. There are the Lowest Level Term (LLT), Preferred Term (PT), High Level Term (HLT), High Level Group Term (HLGT) and System Organ Class (SOC) [38]. Disbiome uses the PT to link the disease to its classification or where more appropriate the LLT. Selecting a certain classification term will give an overview of all the experiments linked to diseases classified in that specific term (e.g. all Gastrointestinal disorders (SOC)). This classification and an overview of all the related experiments linked to a certain disease is presented in the disease detail page.

### Organism

The microbial organisms are classified using the NCBI and SILVA taxonomy and are linked to its corresponding databases. The NCBI taxonomy is the standard nomenclature and classification repository for the International Nucleotide Sequence Database Collaboration (INSDC). It includes organism names and taxonomic lineages for each of the sequences represented in the INSDC’s nucleotide and protein sequence databases [39]. The SILVA database contains taxonomic information of Bacteria, Archaea and Eukarya and is based on small subunit rRNA sequence information [28]. These taxonomies are chosen because NCBI is the most extensive taxonomy (other taxonomies are for the most part contained in the NCBI taxonomy) and goes down to the species level while SILVA’s taxonomic classification is very reliable due to its manual curation. In addition, these databases are updated regularly [40]. An overview of all related experiments linked to a certain organism is given in the organism detail page.

### Detection method

Traditional studies of the microbiome remained largely dependent on cultivation techniques. However, these culture methods are able to detect only 10–30% of the microbiota [41]. Due to rapid development of culture-independent molecular technologies such as PCR-denaturating gradient gel electrophoresis (DGGE), restriction fragment length polymorphism (RFLP), DNA microarray, etc., non-cultivatable organisms could be detected [42–44]. More recently, several next-generation sequencing (NGS) technologies have been developed which make it possible to detect even low abundant micro-organisms [45]. Most recent techniques such as shotgun metagenomic sequencing are able to not only reveal abundancy changes, but also functional changes in the microbiome [41]. The choice of detection method in a microbiome case-control study is of great importance. A NGS method is able to detect certain bacteria where other techniques fail, resulting in different relative proportions of the microbial composition [46]. However, different NGS platforms can produce different microbial profiles. So, it may be necessary to use different platforms to correctly unravel the microbial profile and it is important to use the same platform(s) to make comparisons possible [47].

### Publication

Every experiment in the Disbiome database is linked to the publication of the original research in PubMed. When sequencing data are deposited in a repository, a link to this repository with the accession numbers is given. Due to the rise of NGS technologies, immense amounts of sequencing data are generated. These experimental data should be archived for this is key to the progress of reproducible science. The Sequence Read Archive (SRA) was one of the first archives for sequencing data and was established as a part of the INSDC [48], other data repositories are: GenBank [49], European Nucleotide Archive (ENA) [50] and the DNA Data Bank of Japan (DDBJ) [39]. Additionally, all the data from the Human Microbiome Project (HMP) is freely available through its portal [51].

To ensure that the used methods and results can be reviewed, analyzed and repeated, a minimum amount of relevant information must be included in scientific publications. Numerous standards for conducting and reporting clinical trials have been implemented for years. Examples of these standards are the Cochrane, CONSORT (CONsolidated Standards Of Reporting Trials) [52] and STROBE (STrengthening the Reporting OBservational studies in Epidemiology) initiatives [53]. These measures have improved the reporting quality of clinical trials [54]. In experimental life sciences, such guidelines are implemented more recently. In 2009, Kilkenny et al. performed a survey of the reporting quality of scientific research using animals. This survey identified some issues that need to be addressed in order to improve scientific research [55]. This resulted in the establishment of the ARRIVE reporting guidelines for animal in vivo experiments [56]. Publications in the Disbiome database are assessed for different reporting parameters based on a survey performed by Vesterinen et al. [54]. This survey consists of 16 questions all assessing different aspects of the reporting quality (Table 1). These data are all presented in the publication detail page.

**Table 1:** Questionnaire for assessing reporting parameters of Disbiome publications

Category	Question
Reporting parameters	
1	Is the age of subjects given?
2	Is the geographical origin of study participants given?
3	Are microbiome influencing factors reported (diet, medication, smoking, lifestyle,…)?
4	Is a conflict of interest statement given?
Analysis parameters	
5	Are specific test statistics reported
6	Is a measure of variance (SD, SEM, CI, IQR, boxplot,…) reported?
7	Are numerical microbiome changes given (raw data)?
8	Were numerical data reported for each individual subject?
9	Is the unit of analysis specified?
Design parameters	
10	Is a primary/research hypothesis literally stated?
11	Is a statement about sample/control size given?
12	Are controls matched for possible confounding factors (age, sex, diet,…)?
13	Is type of control group defined?
14	Are inclusion/exclusion criteria stated?
15	Is a statement about sample traceability/history (sampling and storage before analysis) given?
16	Is a statement about sample blinding before analysis given?

## Conclusions

Literature data on microbiome alterations in different disease states is vastly increasing. Disbiome (https://disbiome.ugent.be/) provides an organized overview of this rapidly expanding field of knowledge. Together with the used sample, detection method, methodological details and host information, quantitative data of micro-organisms in patients and controls from a specific experiment are presented. In addition, different reporting parameters of the concerned publications are presented. This is the first database giving a relation between the health status of the host and its microbiota composition.

## Availability and requirements

**Project name:** Disbiome.

**Project home page: **https://disbiome.ugent.be/.

**Browser:** Google Chrome, Microsoft Internet Explorer, Mozilla Firefox.

**Lisence:** none.

**Restrictions for non-academic users:** none.

## Abbreviations

CONSORT: CONsolidated Standards Of Reporting Trials
DDBJ: DNA Data Bank of Japan
DGGE: Denaturating gradient gel electrophoresis
ENA: European Nucleotide Archive
HLGT: Highest level group term
HLT: High level term
ICH: International council for harmonisation of technical requirements for pharmaceuticals for human use
INSDC: International nucleotide sequence database collaboration
LLT: Lowest level term
MedDRA: Medical dictionary for regulatory activities
NGS: Next-generation sequencing
PT: Preferred term
RFLP: Restriction fragment length polymorphism
SOC: System organ class
STROBE: STrengthening the Reporting OBservational studies in Epidemiology
